# Exploring ultrasound and electromyography for carpal tunnel syndrome diagnosis: a comprehensive comparative study and implications for occupational medicine

**DOI:** 10.3389/fneur.2024.1490873

**Published:** 2024-12-11

**Authors:** Salem Braham, Amen Moussa, Marwa Bouhoula, Nihel Ben Meriem, Ichraf Annen, Ghazi Sakly, Asma Chouchane, Malek Ben Abdelkader, Asma Aloui, Imène Kacem, Maher Maoua, Houda Kalboussi, Olfa Elmaalel, Houda Mhabrech, Souheil Chatti, Aicha Brahem

**Affiliations:** ^1^Department of Radiology, Farhat Hached University Hospital, Sousse, Tunisia; ^2^Medical Faculty Ibn Jazzar, University of Sousse, Sousse, Tunisia; ^3^LR19SP03, Occupational Medicine Department, Farhat Hached University Hospital, Sousse, Tunisia; ^4^Department of Neurophysiology, Sahloul University Hospital, Sousse, Tunisia

**Keywords:** carpal tunnel syndrome, ultrasonography, electromyography, notch sign, occupational medicine

## Abstract

**Background:**

To assess the contribution of ultrasound in diagnosing occupational carpal tunnel syndrome (CTS), compare it with electromyography (EMG) results, and evaluate the ultrasound characteristics of CTS patients.

**Methods:**

A nine-month cross-sectional study (January–September 2021) involved CTS patients and a control group, utilizing a structured form for data collection. EMG was performed on the patient group (‘cases’) and ultrasound examinations were conducted on both groups. Statistical analysis was performed using SPSS software.

**Results:**

Among 44 cases and 30 controls, CTS patients (mean age 44.9 years) exhibited predominantly bilateral symptoms (90.9%). The optimal cross-sectional area (CSA) threshold for diagnosis was 10.3 mm^2^ (89% sensitivity, 84% specificity). Significant differences in ultrasound criteria were observed between patient and control groups, including the “notch sign” (*p* = 0.012), hypoechoic appearance (*p* = 0.016), and reduced median nerve mobility (*p* = 0.021). Quantitatively, CSA (13.7 mm^2^ vs. 7.4 mm^2^), flattening ratio (3.3 vs. 2.1), and retinaculum bulging (3.2 mm vs. 1.9 mm) significantly differed between cases and controls (*p* = 0.0019, 0.025, and 0.01, respectively). Positive Phalen tests correlated with higher CSA (*p* = 0.005) and retinacular bulging (*p* = 0.02). CSA correlated with EMG parameters, indicating slower conduction velocities, lower amplitudes, and longer latencies (*p* < 10^(−3), *r* = −0.56, −0.62, −0.36, and −0.68, respectively).

**Conclusion:**

This study highlights ultrasound’s diagnostic potential for CTS, particularly in occupational settings. Its non-invasiveness and reliability advocate for its integration into routine diagnostic protocols, supporting evidence-based management strategies. Further research is needed to explore long-term efficacy and broader applicability.

## 1 Introduction

Carpal tunnel syndrome (CTS) is a frequent compressive neuropathy, predominantly observed in individuals aged 45 to 60, with a higher prevalence among women (1). According to Chammas et al., around 4 to 5% of the global population is estimated to experience CTS (2).

This disorder results from the chronic compression of the median nerve within the carpal tunnel, an osteofibrous structure confined by the carpal bones and the transverse carpal ligament. The median nerve, situated within this confined space alongside the flexor tendons of the fingers, is subjected to increased pressure, leading to CTS (2).

The origins of this condition can vary, ranging from idiopathic CTS with no apparent cause to secondary forms resulting from anatomical, endocrine, infiltrative, tumorous, or infectious factors (1).

Professionally induced factors play an increasingly pivotal role in the upsurge of CTS, particularly in occupations involving repetitive upper limb movements, sustained physical efforts, wrist torsions, and exposure to vibrating tools (3, 4). This has sparked concern in occupational medicine. Luckhaupt et al. reported a lifetime prevalence of 6.7% and a 12-month prevalence of 3.1% (approximately 4.8 million workers) for clinically diagnosed CTS among workers, with 67.1% of cases attributed to work (5). In Tunisia, its prevalence is estimated at 35 cases per 100,000 workers engaged in intensive manual activities (6, 7).

Early and accurate detection of CTS is crucial for adequate management and reducing its impact on individuals’ work capacity (8).

Electromyography (EMG) has traditionally been the gold standard for evaluating median nerve involvement and confirming diagnoses. However, EMG has limitations, including its sensitivity, which can be as low as 56 to 85% (9–11), especially in the early stages of the disease (1, 4). Additionally, EMG can be uncomfortable for patients and may not be readily accessible in all healthcare settings (12).

These challenges have prompted the research for alternative diagnostic methods. In recent years, ultrasound has emerged as a promising tool for CTS assessment. This technology offers significant advantages, including its non-invasive nature, increased availability, and real-time insights into the carpal tunnel’s structure and potential etiological anomalies (12). This has generated growing interest among healthcare professionals, especially in occupational medicine, where occupational CTS cases are rising.

This article aims to examine the contribution of ultrasound in diagnosing occupational CTS, assess its added value compared to EMG, and highlight its potential benefits in improving early detection and management of this condition.

## 2 Materials and methods

This is a cross-sectional study conducted over nine months, spanning from January to September 2021. Participants were selected from patients referred to the Occupational Medicine Department of Farhat Hached University Hospital in Sousse. All participants presented with symptoms evoking CTS according to the criteria of the American Academy of Neurology (9) and whose diagnosis was confirmed by electrophysiological examinations.

All study participants were informed about the research topic. Their participation was contingent upon obtaining free and informed consent, adhering to Tunisian regulations, and aligning with the Helsinki Declaration, and UNESCO’s Universal Declaration on the Human Genome and Human Rights. The study received approval from the Faculty of Medicine Ethics Committee, Sousse (Assessment 244 Reference: CEFMS 244/2024).

Patients who underwent CTS surgery or treatment in the last six months, and those with comorbidities that could cause secondary neuropathy, ongoing pregnancy, or a history of wrist trauma or fracture were excluded due to the lack of clear consensus criteria for the electrodiagnosis of CTS in these conditions, as noted in previous studies (13). A control group, consisting of individuals without any symptoms of CTS, was carefully screened and recruited from the radiology department at Farhat Hached University Hospital in Sousse. These individuals were also subjected to the same exclusion criteria as the case group, including a history of wrist trauma, previous CTS diagnosis, or other conditions that could mimic CTS symptoms.

Data were collected using a structured form that included information on sociodemographic and individual data (age, gender, origin), professional data (sector of activity, occupation, occupational seniority, specific tasks), medical data (personal history, functional signs, clinical examination data, and medications), as well as ultrasound and EMG data.

EMG examinations were conducted for the patients, by the same operator, in the Neurophysiology Department at the University Hospital Sahloul in Sousse. They were performed using a Neurosoft-MEP 4 device, with parameters including nerve conduction velocity, sensory response amplitude, distal motor latency, and muscle response amplitude. The severity of CTS was classified into six grades according to the Bland criteria (14).

All ultrasound examinations were performed using a Philips iU22 ultrasound machine equipped with a linear multifrequency L12-5 50 mm (5–12 MHz) probe. The same operator conducted all examinations on both groups at the Radiology Department of Farhat Hached Hospital in Sousse. Patients were positioned comfortably with their forearms resting on the table, palms up, in a neutral position. The palmar crease of the wrist, along with carpal bone landmarks, was used as a reference point for probe placement. Both transverse and longitudinal planes were used to evaluate the entire course of the median nerve within the carpal tunnel. To ensure consistency, all ultrasound examinations were conducted by the same operator.

A single-blind design was employed to minimize diagnostic bias. Operators conducting the ultrasound examinations were blinded to the patient’s clinical status and diagnosis by using a coding system that concealed patient information until after the examinations were completed. A centralized data management system was implemented. Patient information was coded and kept confidential throughout the study, preventing operators from accessing clinical details before or during the ultrasound examinations. Regular quality control checks were conducted to verify the integrity of the blinding process. Additionally, data analysis was performed by individuals who were unaware of the patient groups, further reinforcing the blind design.

The study focused on the analysis of ultrasound criteria, including the CSA of the median nerve, the flattening ratio, the bulging of the retinaculum, the notch sign, changes in the echotexture of the median nerve, and decreased mobility during flexion-extension.

Statistical analysis of the data was performed using SPSS software version 25.0. Normality tests (Kolmogorov–Smirnov or Shapiro–Wilk) confirmed that all data followed a Gaussian distribution (*p* > 0.05). This allowed for the use of parametric tests, such as Student’s t-test, to compare continuous variables (age, weight, height, BMI, CSA at the inlet, evolution duration of the symptoms, and CSA at the outlet) between patients and controls.

Gender and involved sides, being categorical variables, were analyzed using a chi-square test. Pearson’s correlation, a parametric test suitable for analyzing the relationship between continuous variables, was used to assess the relationships between CSA at the tunnel inlet and weight, height, and BMI. A significance level of *p* ≤ 0.05 was adopted for all statistical analyses.

Receiver operating characteristic (ROC) curves were used to determine optimal cutoff points, assess sonographic measurements’ diagnostic accuracy, and depict sensitivity and specificity variations with different cutoff points. Data from both right and left wrists were analyzed cumulatively to increase the sample size and enhance the statistical power of the analysis. This approach also acknowledges that CTS can often affect both wrists symmetrically.

To assess differences in sensitivity and specificity, the exact McNemar test, a non-parametric test suitable for paired categorical data, was employed.

## 3 Results

A total of 44 cases and 30 controls participated in this study. The mean age of patients was 44.9 ± 7 years (range, 32–55 y), and controls had a mean age of 42.5 ± 11 years (range, 28–58 y).

The majority of patients in our study were employed in manufacturing (45.5%), followed by healthcare (25%). Common job positions included blue-collar workers (36%), seamstresses (18%), and nurses (13.6%). The occupational seniority of patients averaged 21.1 years (standard deviation: 6.2).

The average duration of symptoms in the patient group was 19 months (range, 3–120 mo). The majority of cases (90.9%) experienced bilateral involvement, and four patients had unilateral involvement. Consequently, we analyzed a total of 84 wrists in the ‘case’ group. Common symptoms included numbness, painful discomfort, paresthesia, and hand weakness, localized in the median nerve territory in 75% of cases. Clinical signs such as Phalen, Tinel, and McMurtry signs were noted in the majority of cases (Table 1).

**Table 1:** Sociodemographic and clinical characteristics of the cases and controls.

Characteristic	Patients (*N* = 44)	Controls (*N* = 30)
Age, years, mean (range)	44.9 (32–55)	42.5 (28–58)
Sex, *n* (%)		
Man	15 (34)	15 (50)
Woman	29 (66)	15 (50)
BMI, Kg/m^2^, mean (range)	29.22 (22.4–39.4)	27.35 (23.6–34.2)
Work seniority, years, mean, SD	21.1 ± 6.2	-
The average duration of symptoms, Months, mean, SD	19 (3–120)	-
Clinical signs, *n* (%)		
Bilateral	40 (90.9)	-
Phalen Sign	41 (94)	-
Tinel Sign	39 (88.1)	-
McMurtry	34 (78.6)	-
Hyposthesia	12 (27.4)	-

SD, Standard deviation.

An analysis was conducted to explore correlations between ultrasound parameters and demographic characteristics. ROC curves identified an optimal threshold for the CSA at 10.3 ± 0.02 mm^2^, with a sensitivity of 89% and specificity of 84%. For the flattening ratio, the threshold was 2.35 ± 0.33, with a sensitivity of 72% and specificity of 78%. The retinaculum bulging threshold was approximately 3.15 ± 0.54 mm, with a sensitivity of 65% and specificity of 80%. The CSA proved to be the most sensitive and specific sign. Various qualitative signs showed specificity values ranging from 72 to 78%, with the notch sign being the most sensitive at 80% (Table 2).

**Table 2:** Diagnostic metrics for ultrasound criteria: specificity vs. sensitivity.

Ultrasound criteria	Sensitivity (%)	Specificity (%)
CSA > 10.3 mm^2^	89	84
FR > 2.35	72	78
RB >3.15 mm	65	80
i. Notch sign	80	78
ii. Hypoechoic appearence	77	75
iii. Decreased mobility	43	72

CSA, Cross-sectional area Values are rounded; FR, Flattening ratio; RB, Retinacular bulge.

There was no correlation between the CSA and age (*p* > 0.05), BMI (*p* > 0.05), or gender (*p* > 0.05).

Ultrasound criteria demonstrated significant distinctions between patient and control groups, with the “notch sign” appearing in 71.4% of patients versus 6% in controls, hypoechoic appearance in 77.4% of patients versus 16% in controls, and reduced median nerve mobility during flexion-extension in 42.9% of patients and 6% of controls (*p* = 0.012, 0.016, and 0.021, respectively). Quantitatively, significant differences were observed in the median CSA (13.7 mm^2^ vs. 7.4 mm^2^), flattening ratio (3.3 vs. 2.1), and retinaculum bulging (3.2 mm vs. 1.9 mm) between cases and controls (*p* = 0.0019, 0.025, and 0.01, respectively) (Table 3).

**Table 3:** Ultrasound characteristics in both groups.

Ultrasound characteristic	Total wrists studied in cases (*N* = 84)	Total wrists studied in controls (*N* = 60)	*p*
Notch sign, *n* (%)			
No	24 (28.6)	58 (96.6)	0.012
Yes	60 (71.4)	2 (3.3)	
Hypoechoic appearance, *n* (%)			
No	19 (22.6)	50 (83)	0.016
Yes	65 (77.4)	10 (16)	
Decreased mobility of the median nerve during flexion-extension movements, *n* (%)			
No	48 (57.1)	58 (96.6)	0.021
Yes	36 (42.9)	2 (3.3)	
CSA at the inlet, mm^2^, threshold	13.7 [7–24]	7.4 [6–14]	0.0019
The median flattening ratio, mean	3.3 [1.5–6]	2.1 [2–4.5]	0.025
The median retinaculum bulging (mm^2^), mean	3.2 [2.1–5.5]	1.9 [1.8–4.5]	0.01

A statistically significant, moderate positive correlation (*r* > 0) was observed between the duration of CTS and the CSA (*p* = 0.04), flattening ratio (*p* = 0.021), and retinaculum bulging (*p* = 0.025) (Table 4).

**Table 4:** Correlation between symptom duration and ultrasound findings in CTS.

	<1 month	1–12 months	>12 months	*p*
Cross sectional area (mm^2^)	13 ± 4.1	16 ± 3.8	18.5 ± 5.4	0.04
Flattening ratio	2.4 ± 1.8	3.5 ± 1.6	5.2 ± 1.5	0.021
Retinaculum bulging (mm)	2.8 ± 1.1	3.7 ± 1.4	4.8 ± 2.1	0.025

However, no statistically significant relationship was observed between the duration of CTS evolution and various qualitative ultrasound signs (*p* > 0.05).

We conducted independent comparisons of mean values for various quantitative indicators based on the presence or absence of clinical signs. Positive Phalen test results exhibited statistically significant associations with higher values in both CSA (*p* = 0.005) and retinacular bulging (*p* = 0.02). Similarly, hypoesthesia and hand swelling demonstrated statistically significant correlations with an increased CSA (*p* = 0.004, *p* = 0.03 respectively) and retinacular bulging (*p* = 0.016, *p* = 0.021 respectively) (Table 5).

**Table 5:** Relationship between quantitative ultrasound signs and clinical signs.

Clinical sign		Cross-sectional area		Flattening ratio		Retinacular bulge	
		Mean	*p*	Mean	*p*	Mean	*p*
Tinel’s sign	Yes	14.11	NS	3.18	NS	3.32	NS
	No	13.14		2.98		3.12	
Phalen’s sign	Yes	14.22	0.005	3.12	NS	3.1	0.02
	No	9.6		3.01		2	
McMurtry sign	Yes	14.09	NS	3.21	NS	2.98	NS
	No	13.2		2.67		2.85	
Median nerve hypoesthesia	Yes	15.55	0.004	2.94	NS	2.67	0.016
	No	11.16		3.16		3.18	
Muscle atrophy	Yes	13.89	NS	2.99	NS	2.8	NS
	No	13.51		3.11		2.7	
Hand swelling	Yes	18.25	0.03	3.63	NS	2.7	0.021
	No	13.64		3.02		3.7	

Regarding qualitative ultrasound signs, we found a significant association between the Phalen test and the notch sign (*p* = 0.004), the latter being more prevalent with a positive Phalen test. Carpal canal content mobility decrease was more frequent in patients with a positive Phalen test (*p* = 0.002). Additionally, a significant association was found between hypoesthesia in the median nerve territory and the ultrasound notch sign (*p* = 0.009). No significant correlations were identified between the Tinel sign, McMurtry sign, muscle atrophy in the thenar compartment, hand swelling, and various ultrasound signs (Table 6).

**Table 6:** Relationship between qualitative ultrasound signs and clinical signs.

Clinical sign		Notch sign		Hypoechoic appearance		Decrease mobility	
		%	*p*	%	*p*	%	*p*
Tinel’s sign	Yes	71.6	NS	77	NS	56.7	NS
	No	70		80		40	
McMurtry sign	Yes	73.4	NS	79.7	NS	74.8	NS
	No	56		40		54	
Phalen’s sign	Yes	78.8	0.004	81.8	NS	61.5	0.002
	No	24.4		61.1		11	
Medial nerve hypoesthesia	Yes	91.6	0.009	95.8	0.011	58.3	NS
	No	23.3		25.4		57.8	
Muscle atrophy	Yes	92	NS	92	NS	53.8	NS
	No	74		75		40.8	
Hand swelling	Yes	71.6	NS	100	NS	66.6	NS
	No	66		76		42	

Significant correlations were found between ultrasound signs (CSA, flattening ratio, and retinaculum bulging) and EMG parameters, indicating moderate relationships. Higher CSA values were associated with slower sensory conduction (*p* < 10–3, *r* = −0.56), and motor conduction velocities (*p* < 10–3, *r* = −0.62), lower sensory action (*p* < 10–3, *r* = −0.36) and motor action potential amplitudes (*p* < 10–3, *r* = −0.68), and longer distal motor latencies (*p* > 10–3, *r* = 0.55) (Table 7).

**Table 7:** Relationship between the CSA of the NM and the EMG parameters.

Parameter	*p*	*r*
SCV*	<0.001	−0.56
MCV	<0.001	−0.62
SAP	<0.001	−0.36
MAP	<0.01	−0.68
DML	<0.001	0.55

*SCV, MCV: sensory and motor nerve conduction velocities. SAP, MAP: amplitude of sensory and motor action potential. DML: distal motor latency.

## 4 Discussion

Carpal Tunnel Syndrome (CTS) is a prevalent and clinically significant condition, often challenging to diagnose accurately. Our cross-sectional study aimed to enhance the understanding of CTS by comparing the diagnostic accuracy of ultrasound with nerve conduction studies and electromyography. We also sought to evaluate the ultrasound characteristics of CTS patients and compare these findings with electromyography characteristics. The results revealed differences and associations that provide valuable insights into the diagnostic aspect of CTS.

Clinical signs, including the Phalen, Tinel, and McMurtry signs, were substantially present in the patient group compared to the controls, reflecting common diagnostic features observed in this population.

While clinical provocation tests have demonstrated low positive predictive values, sensitivity, and specificity, the diagnosis of CTS traditionally relies on the association of clinical data and electrophysiological studies (15).

Electrophysiological tests, although specific, exhibit significant variability in sensitivity (16). This variability poses challenges in achieving consistent and reliable results. Addressing these challenges, ultrasound has emerged as a promising diagnostic modality, offering potential advantages in terms of sensitivity and precision. This is evidenced by several studies comparing it to electromyography in diagnosing CTS (17–21).

Our study highlighted a significant relationship between the duration of symptom evolution and ultrasound metrics, showing that alterations in ultrasound findings became more important as the symptoms persisted. This is in line with previous studies, such as that by Filho A-G et al. (22) and Chen S-F et al. (23), which also found a correlation between symptom duration and the CSA of the median nerve measured by ultrasound.

It is important to note that in our study, there was no statistically significant relationship between age and different quantitative and qualitative ultrasound parameters. However, other studies have shown that age can impact the severity and clinical presentation of CTS, with more severe electrophysiological results in older patients (24–26). This has been explained by the fact that the pathophysiological response to chronic nerve compression varies with age. Indeed, in cases of severe long-term compressive neuropathies, nerve atrophy, and axonal degeneration have been described. This is seen more in the older adult who reach terminal stages of the disease characterized by nerve atrophy associated with fibrotic changes (26). Additionally, the inflammatory response and the capacity for edema formation may be limited in older adult individuals. Therefore, it can be assumed that the degree of median nerve swelling at the entrance of the carpal canal may be reduced in older patients with moderate to severe CTS (25–28).

Several researchers have emphasized that CTS is more common in the obese population, with a two to three-times higher risk of being diagnosed with CTS (29). However, our study found no statistically significant relationship between ultrasound parameters, especially the nerve CSA and BMI. This is consistent with other research, such as those by Roghani R et al. (30) and Hunderfund ANL et al. (13), who did not find a statistically significant correlation between BMI and nerve CSA.

Our study revealed a predominance of females in the sample. The correlation between the female gender and the risk of developing CTS is well documented in the literature. However, our study did not find a significant correlation between gender and ultrasound parameters, which is in line with several other studies in the literature that did not find an influence of gender on the nerve CSA or other ultrasound parameters (7–9, 12).

Our findings align with previous studies in demonstrating a strong correlation between clinical signs, particularly Phalen’s, Tinel’s, and McMurtry’s tests, and ultrasound parameters in diagnosing CTS. Studies by Kotevoglu et al. (31), Filho et al. (22), and El-Najjar et al. (32) have similarly shown significant associations between these clinical tests and ultrasound findings. Moreover, Naranjo et al. (33) highlighted the superior diagnostic value of ultrasound compared to physical maneuvers alone, supporting our findings. These collective results underscore the complementary nature of clinical examination and ultrasound and the diagnostic value of ultrasound, particularly in occupational settings where early detection and intervention are crucial.

Ultrasound evaluation in our study revealed significant differences between the case and control groups. Notable differences in the notch sign, hypoechoic appearance, and reduced median nerve mobility during flexion-extension movements were observed. However, the inherent variability of qualitative parameters remains a limitation, even with standardized examinations by a single operator. Ferrari FS et al. showed an average sensitivity of 50% for detecting changes in the echo structure of the median nerve in ultrasound (34). Other studies, such as that of Wang L-Y et al., identified the notch sign as an important ultrasound parameter after the CSA (35). Recently, a study conducted by Akturk S et al. (36), on 41 patients and 20 controls, revealed significant decreases in echogenicity and mobility in patients compared to controls. These results are consistent with other studies that observed a reduction in median nerve mobility under the retinaculum during finger flexion or extension in CTS patients (37, 38). However, it is important to note that this feature remains subjective and difficult to quantify, as highlighted by Sarría L et al. (39).

Furthermore, in this study, the CSA of the median nerve at the carpal tunnel inlet emerged as a significant metric, significantly larger in CTS patients compared to the control group. This aligns with previous research by Buchberger et al. (40), describing anatomical changes visible in ultrasound. They suggested that the thickening of the nerve at the proximal part of the canal and its non-physiological flattening at the distal part are the two most reliable parameters for ultrasound diagnosis of CTS. As per the literature, various diagnostic criteria have been proposed, and the main ultrasound sign in favor of CTS is the thickening of the median nerve evidenced by an increase in the value of its CSA in its intra-canal course (41). However, there is currently no consensus on the threshold value of the CSA for a positive diagnosis. Pathological thresholds vary between 9 mm^2^ and 15 mm^2^ according to different studies. In this study, an optimal threshold value of 10.3 mm^2^ was found, providing a sensitivity of 89% and a specificity of 91%. The sensitivity and specificity of ultrasound have been assessed in various studies, showing varied results. For example, one study found a sensitivity of 67% and a specificity of 97% for ultrasound in the diagnosis of CTS, comparable to EMG (42). Another suggested a CSA threshold of 10 mm^2^ for a sensitivity of 82% and a specificity of 87% (43). A Tunisian study showed a sensitivity of 93% for the diagnosis of CTS by ultrasound (37).

The variability in results between these studies can be attributed to several factors, such as patient and control selection criteria, the standard reference for CTS diagnosis, electrodiagnostic methods, and differences in ultrasound techniques, including nerve CSA measurement levels (44, 45).

Visser et al. found that, among 28 patients with negative EMG, 16 had positive sonograms (17). Sonography showed 78% sensitivity and 91% specificity, while EMG tests for Distal motor latency of the (DML) median nerve >3.8 msec demonstrated 74% sensitivity and 97% specificity. Additionally, according to Kele et al., ultrasound demonstrated a higher predictive value with a CSA > 0.11 cm^2^, showing 89.1% sensitivity and 98% specificity (46). This aligns with our study, reinforcing the utility of ultrasound in assessing nerve health with notable sensitivity and specificity values.

As previously noted, ultrasound offers significant advantages, including its low cost, portability, non-invasive nature, and real-time insights into the carpal tunnel’s structure and potential etiological anomalies (12). These characteristics make ultrasound a valuable tool for early detection and prevention of CTS in occupational settings.

To further enhance the relevance of this study in occupational health, it is important to highlight the specific types of jobs that are most affected by CTS. Studies have consistently identified occupations involving repetitive hand movements, forceful gripping, and awkward postures as significant risk factors for CTS (3, 4). Examples of high-risk industries include manufacturing, healthcare, and office work, particularly jobs involving computer use for extended periods.

Incorporating ultrasound as a regular diagnostic tool in these high-risk occupational settings could significantly improve early detection and prevention of CTS. This could involve conducting ultrasound screenings for employees in targeted job roles, especially those with a history of wrist symptoms or those who engage in repetitive hand movements. Early identification of CTS would enable timely intervention, such as ergonomic adjustments, physical therapy, or in severe cases, surgical treatment, thereby reducing the impact of the condition on employee health and productivity.

The implementation of ultrasound in workplace health policies or routine screenings could be a valuable tool for preventing severe complications, absenteeism, and productivity decline associated with CTS (8). According to Karabeg et al., the non-invasive nature of ultrasound aligns seamlessly with the primary goal of occupational health and safety—ensuring employee well-being and maintaining optimal productivity levels (8).

The present study has both strengths and limitations. Among its strengths, this study stands out from previous ones by adopting a case–control approach, unlike many studies that have mainly focused on symptomatic patients. Furthermore, the study goes beyond the simple evaluation of the CSA of the median nerve in the carpal canal by including quantitative and qualitative measurements, thus providing a more comprehensive diagnostic contribution. Operators performed both ultrasounds and electromyography, reducing inter-observer variability, while the short interval between the two examinations minimizes potential influences on the results. Moreover, the sample size is adequate for reliable statistical analysis. Participant selection criteria were carefully chosen to minimize selection biases. Finally, the implementation of a ‘single-blind’ approach in our study, wherein operators conducted examinations without prior knowledge of patients’ clinical information, adds a robust layer to the methodology, minimizing potential biases and enhancing the objectivity of the findings.

However, the study has some limitations. Although asymptomatic controls were included, electrodiagnostic were not performed on these subjects, limiting the comparison to symptomatic patients. Furthermore, the comparison between imaging data and electrodiagnostic data is complex due to the lack of imaging diagnostic standards.

In summary, our cross-sectional study highlights the significant diagnostic potential of ultrasound in CTS. By correlating clinical manifestations with ultrasound metrics, we identified associations and differences, emphasizing the value of a multimodal diagnostic approach. The study supports the inclusion of ultrasound in routine diagnostic protocols, particularly in an occupational context, where early intervention could mitigate complications. While recognizing the study’s strengths, we acknowledge limitations and advocate for further research to explore the long-term efficacy and broader applicability of ultrasound in CTS diagnosis. This work contributes to advancing evidence-based strategies for the effective management of CTS in at-risk populations.

## Data Availability

The datasets presented in this study can be found in online repositories. The names of the repository/repositories and accession number(s) can be found in the article/supplementary material.
